# Incremental and transformational climate change adaptation factors in agriculture worldwide: A comparative analysis using natural language processing

**DOI:** 10.1371/journal.pone.0318784

**Published:** 2025-03-19

**Authors:** Sofia Gil-Clavel, Thorid Wagenblast, Tatiana Filatova

**Affiliations:** Department of Multi-Actor Systems, Faculty of Technology, Policy, and Management, Delft University of Technology, Jaffalaan, Delft, The Netherlands; Politecnico de Milano, SPAIN

## Abstract

Climate change is projected to adversely affect agriculture worldwide. This requires farmers to adapt incrementally already early in the twenty-first century, and to pursue transformational adaptation to endure future climate-induced damages. Many articles discuss the underlying mechanisms of farmers’ adaptation to climate change using quantitative, qualitative, and mixed methods. However, only the former is typically included in quantitative metanalysis of empirical evidence on adaptation. This omits the vast body of knowledge from qualitative research. We address this gap by performing a comparative analysis of factors associated with farmers’ climate change adaptation in both quantitative and qualitative literature using Natural Language Processing and generalized linear models. By retrieving publications from Scopus, we derive a database with metadata and associations from both quantitative and qualitative findings, focusing on climate change adaptation of farmers. We use the derived data as input for generalized linear models to analyze whether reported factors behind farmers’ decisions differ by type of adaptation (incremental vs. transformational) and across different global regions. Our results show that factors related to adaptive capacity and access to information and technology are more likely to be associated with transformational adaptation than with incremental adaptation. Regarding world regions, access to finance/income and infrastructure are uneven, with farmers in high-income countries having an advantage, whereas farmers in low- and middle-income countries require these the most for effective adaptation to climate change.

## Introduction

The impact of climate change on agriculture already has negative consequences for food security [1,2], populations [2,3], and economies [3,4]. The Intergovernmental Panel on Climate Change projects that climate change will likely negatively impact average global crop yields as soon as 2030 [1], with already declining yields in Africa [5], South-Asia [6], and the UK [3] threatening food security. In terms of countries’ populations, the negative effects on agriculture and resulting undermined livelihoods are expected to exacerbate societal problems, such as poverty, health, and unemployment [2,3]. Economically, the shortfalls in countries’ agricultural production are expected to create unemployment, exhaust global stocks, and skyrocket food prices [3,4,7].

These adverse climate-induced effects can be ameliorated if farmers start adapting, at least incrementally earlier in the twenty-first century, or switch to transformational adaptation during the second half of the century [8]. Incremental Climate Change Adaptation (CCA) is a gradual adjustment to actual or expected climate and its effects by conventional means [1]. In the agricultural context, incremental CCA involves changes in seed varieties, planting times, or upgrades of existing irrigation. Conversely, transformational CCA entails unprecedented scaling of conventional measures, an introduction of novel measures not previously practiced locally, or relocations of activities to new places [9]. In the agricultural context, transformational CCA may include migration, crop relocation, or changing the farming system, such as shifting from irrigated to dryland [8]. While both incremental and transformational adaptations are essential, the factors associated with their adoption may differ.

To date, several literature reviews systemize information on what constitutes farmers’ adaptation measures [10–12], and what factors possibly drive their uptake [13–17]. Typically, such reviews are limited to meta-analyses of quantitative survey data [17,18] or cover a limited number of qualitative and quantitative articles that can be processed manually [19]. Growing empirical evidence on adaptation makes it increasingly difficult to extract information from the rich literature and to update such knowledge databases. Automizing this process is important for the timely analysis of large amounts of data and for eliciting trends [20]. It is in this context that computer science tools, like Machine Learning (ML), add value. As such, ML is used to identify CCA-relevant publications [20,21] and to track where and how CCA is progressing [21]. This is done by training classifiers to help speed-up filtering out irrelevant articles, and/or to detect and assign variables’ values from textual data [20,21]. However, ML falls short in eliciting nuanced information on relations between CCA measures and different economic, demographic, and psychological factors. This is where tools like Natural Language Processing (NLP) can be effectively utilized.

NLP encompasses various computational techniques that help machines understand human language by grasping its meaning. This involves not only recognizing the statistical characteristics of language (e.g., word count) but also understanding the meaning and context of individual sentences [22]. Its application in extracting relations of connected concepts is particularly valuable for our goals. Relation extraction involves identifying and categorizing semantic relationships from text, such as whole–part, product–producer, and cause–effect relationships [23]. This technique is widely used in fields like medicine, where extracting causal relationships from medical literature helps build knowledge graphs. These knowledge graphs enable experts to swiftly identify causal links, such as diseases causing symptoms, diseases leading to complications, treatments improving conditions, and ultimately tailoring treatment plans [23]. Despite NLP great advantages, to our knowledge, NLP for relation extraction has seldom been used in the field of CCA [24].

Leveraging the advances in machine learning and NLP, we review the growing empirical evidence on CCA factors. We explore whether factors influencing agricultural farmers’ (hereafter referred to as farmers) CCA vary depending on the type of adaptation (incremental vs. transformational) and whether these CCA factors differ across regions worldwide. Going in-depth into the articles’ findings rather than only abstracts, we reveal relationships between CCA measures and the factors associated with these adaptations. Our analysis aims to answer the following research questions: Are there factors that are more likely to be associated with transformational adaptation compared to incremental adaptation? Do the identified adaptation factors vary across different world regions?

To answer these questions, we first select the relevant data by performing active learning analysis on articles retrieved in August 2022 from Scopus [25]. Active learning is a type of ML where models are trained by constantly providing human feedback on hand-checked data points. This enables the algorithm to learn, drastically reducing the total number of articles that require manual screening [26]. Methodologically, we propose a novel way to study articles from different disciplines, regardless of whether they use qualitative, quantitative, or mixed methods. Our methodology relies on using NLP to derive a database of interlinked concepts identified as adaptation factors. We then use this database as an input to generalized linear models to study how these factors associate with different types of CCA (incremental and transformational), and, whether CCA factors vary globally.

This article contributes to the literature in two ways. First, we introduce a methodology that allows researchers to standardize and quantitatively compare findings from quantitative and qualitative articles in an automatized way, revealing relationships between studied concepts rather than simply counting them. Second, we reveal which factors are more likely to be associated with farmers’ adaptation (incremental/transformational) and the regional variation in these patterns. The presented approach can support policy design by generalizing empirical information across cases on what, and where, encourages farmers’ adaptation to climate change. In the following sections, we first describe how we extract the articles’ findings from which we then distill CCA measures and factors. Next, we use these variables to construct a network of concepts with connections constructed based on reported relationships. We then explain the type of generalized linear models used to answer our research questions. Lastly, we discuss how our results contribute to the general understanding of how farmers adapt to climate change and highlight what policymakers can do to encourage farmers to implement additional adaptation measures.

## Background: Understanding farmers’ autonomous adaptation to climate change

The approach introduced in this article is generic and can be used across a range of applications. To contextualize it for our problem at hand – i.e., understanding what affects farmers pursuing transformational or incremental CCA – we provide details on these actions and factors. These CCA measures and factors are used as umbrella terms for applying the NLP algorithms to extract knowledge from the textual data and to construct our network of interrelated concepts. In this context, a CCA measure is an action that a farmer could undertake to reduce current or future losses from adverse effects of climate change. A factor is a driver or a barrier associated with farmers’ adaptation measures as reported in the published literature; they serve as dependent variables in our analysis. We further explain these concepts in the following subsections.

### Farmers’ climate change adaptation measures

To reduce the adverse consequences of climate-induced hazards, farmers can take various measures. In terms of measures, following the literature reviews by Bahinipati et al. [10], Below et al. [11], and Shaffril et al. [12], we categorize farmers’ CCA options into: (i) Crop Management, (ii) Irrigation and Water Management, (iii) Farm-Management, (iv) Financial-Management, (v) Physical Infrastructure Management, and (vi) Information Management. The first two categories are typical responses to severe droughts and to hot and dry summers; these measures together contribute to addressing the adverse consequences of the decline of average annual rainfall [12]. Specifically, *Crop Management* encompasses crop adaptation options, such as crop diversification and relying on improved crop varieties, like drought-resistant crops (S1 Appendix). These adaptation measures help farmers minimize the risks associated with productivity and income loss by directly reducing climate-induced crop losses [10]. CCA in the form of *Irrigation and Water Management* denotes the implementation of measures like irrigation, water conservation, micro irrigation, supplementary irrigation, irrigation in general, and other water management approaches to reduce water shortages [11].

CCA in the form of *Farm-Management* encompasses adjustments in land and livelihood strategies that go beyond agricultural practices [10–12]. Examples include soil conservation, insurance schemes, tree planting, agroforestry and organic farming, in-house farming, and other farm management practices. The adoption of these CCA measures might require new skills. Farmers typically adopt Farm-Management measures when the number of extreme events per year increases, where it becomes important to have a new or diversified response to these adversities [12]. For example, soil conservation could be a response to soil erosion caused by (among others) increased rainfall [12]. The adoption of insurance for crop failure and property loss helps farmers access immediate cash. Some Farm-Management measures have co-benefits, like switching to tree planting, since trees have shorter recovery periods after most natural hazards and provide extra protection against landslides [12].

*Financial-Management* refers to farm-level income strategies that reduce the risk or ameliorate the effect of climate-related income losses [10,11]. Such CCA responses include income diversification including non-farm activities, relying on livestock or fish rearing, taking loans, spending savings, reducing household expenditures, and selling off belongings [12]. Non-farm activities range from opening a small grocery store to employment in other industries, such as construction, nursery production, sales, security, and local commercial production [10,12]. Relying on livestock or fish rearing helps farmers increase their economic liquidity. Another CCA strategy, named *Physical Infrastructure management*, refers to all measures where physical infrastructure is updated to become climate-resilient [12]. This could be improving road conditions, communication and irrigation systems that directly impact farm business. These measures also consider structural modifications made to farmers’ houses and farms, such as wall protection made from bricks, cement, and iron rods to protect against landslides.

Finally, *Information Management* as a CCA action comprises knowledge acquisition and information exchange via peer networks [10–12]. Knowledge management includes the adoption of local knowledge, practical training for farmers and agricultural extension officers, the use of decision support systems and weather forecasts, and wild plants and animals as bellwethers of ecosystem variability or change. Peer networks involve the exchange of CCA practices and experience via family ties and social networks, collective provision of farm inputs, collective marketing of farm products, farmer-to-farmer training, and establishing barter systems. This CCA category could include out-of-farm migration [12], typically to cities where rural inhabitants already know somebody from their social network.

### Incremental vs. transformational adaptation measures among farmers

The fast pace at which climate change is affecting humanity has created the necessity to move from incremental (previously referred to just as adaptation) to transformational adaptation [27,28]. Incremental CCA includes efforts to act through technical and standardized means within the boundaries of existing systems. This usually generates small-scale gradual alterations to existing practices and conventions via marginal changes, in other words, business as usual with minor adjustments. For instance, the introduction of fertilizers or changing crops from wheat to maize serve as examples of incremental changes to existing practices as they require no large-scale restructuring and are focused on short-term effects.

Transformational CCA is often viewed as a process where rules and norms are shifting, enabling new development trajectories to emerge. It requires fundamental changes in the system to address the problems underlying the vulnerability [29], potentially causing massive changes in land use, society, and climate [30]. It includes adopting a future-oriented approach, which acknowledges the need for speed, uncertainty, resilience, and learning in adaptation. Kates et al. [9] define three classes of transformational CCA. First, transformational adaptations are applied at a much larger scale or intensity, for example, the large-scale adoption of farmer-managed natural regeneration in Niger [9]. Second, transformational CCA includes actions that are truly new to a region/system, like introducing new seeds or crop insurance against weather extremes to regions where they were not used before. Third, transformational CCA may include massive land use changes to places and shifting locations of activities, like relocation and migration. Vermeulen et al. [31] offer a more quantitative definition: transformational adaptation is a drastic change happening in response to climate risks, which entails a redistribution of at least a third of the primary factors of production and/or outputs and outcomes of production within 25 years. Their examples of transformational CCA via farm and crop management include moving from agriculture to other types of farming that demand drastic changes in activities, such as aquaculture or orchard farming.

The difference between incremental and transformational adaptation is not clear-cut and depends not only on the scale of analysis (e.g., time, space) [9] but also on the cumulative effects of individual actions [32]. An adaptation that is new to one region (and hence transformational), could be a longstanding practice (i.e., incremental) in another, calling for region-specific analysis. Kates et al. [9] give the example of crop insurance: it has long been done in developed countries (i.e., incremental), but its introduction in some African countries is new (i.e., transformational). Based on this literature review, we classify the collected CCA measures as either incremental or transformational (S1 Appendix).

### Factors associated with farmers’ adaptation measures

Factors facilitating or hindering farmers’ CCA are well studied via surveys, interviews, focus group discussions, serious games, and other social sciences methods. While economic factors do play a role in enabling or hindering adaptation, other socio-behavioral factors intervene making these choices “boundedly rational.” Explanations of farmers’ CCA include: *demographic and socio-economic factors; cognitive and psychological factors; experience of natural hazards; resources, services, and technologies; institutional and political factors*; and *social and cultural factors* [13,15,17].

*Demographic and socio-economic factors* may define farmers’ willingness to adopt new technologies. For example, farmers’ age and gender are related to their willingness to take risks, with young and male farmers more likely to be risk-takers [13]. Gender is also associated with farmers’ likelihood to adopt transformational adaptations, where masculinity-dominant settings may play a role [15]. Education is commonly considered an enabler of adaptation, as education facilitates access to information and the adoption of new technologies [13].

*Cognitive and psychological factors* are important enablers of adaptation in general [17]. In the case of farmers, climate-skeptical farmers are less likely to undertake CCA [16]. In that sense, social capital to conduct adaptation measures is considered an important driver of adaptation [13,17], where social capital might be especially important for farmers’ transformational adaptation [15]. *Experience of natural hazards* is also reported as a driver of adaptation (or maladaptation) [15,17,27]. Specifically, drought and precipitation variability are important motivators of CCA for African and Latin American farmers [27]. Furthermore, *resources, services, and technologies* determine the CCA type that farmers undertake. For instance, access to agricultural extension facilitates crop diversification and planting trees, while access to credit is associated with undertaking soil conservation, irrigation, and changing planting dates [13].

While the abovementioned factors are enablers of adaptation, there are others that could drive or deter adaptation, such as formal *institutional and political factors* as well as informal *social and cultural factors*. In the case of *institutional and political factors*, the relevance and direction of their association with farmers’ adaptation depends on the country [13,16]. Sometimes, non-governmental organizations support farmers in adaptation strategies whilst state institutions do not. As such, governmental directives (or lack of them) can become a major driver or constraint of farmers’ (transformational) adaptation, together with acceptance of (un)favorable government policies or lack of trust in state institutions [13,15]. Lastly, *social and cultural factors* can deter individuals from adopting certain behaviors as they are seen as inappropriate according to their culture and social norms [13,15]. Moreover, in some cultures, depending on the status, individuals are trapped in certain practices as they are not allowed to seek alternative livelihoods [13]. While factors and their associations with CCA in urban settings vary across countries [18], a systematic analysis regarding cross-cultural differences in farmers’ adaptation factors is lacking.

## Methods

To answer the research questions, we apply NLP and multinomial models to perform a comparative analysis of farmers’ CCA factors, differentiating between incremental and transformational adaptation and among world regions. Specifically, we use an interpretable NLP algorithm [33] to extract information on factors associated with farmers’ adaptation measures (as classified by Bahinipati et al. [10], Below et al. [11], and Shaffril et al. [12], see above).

### Systematizing factors associated with farmers’ climate change adaptation

To classify farmers’ CCA factors, we use the interpretable algorithm together with descriptive visualizations to summarize the reviewed articles’ findings [34]. Here, the goal is to extract possible factors associated with farmers’ CCA reported in the articles’ findings.

**Fig 1:**
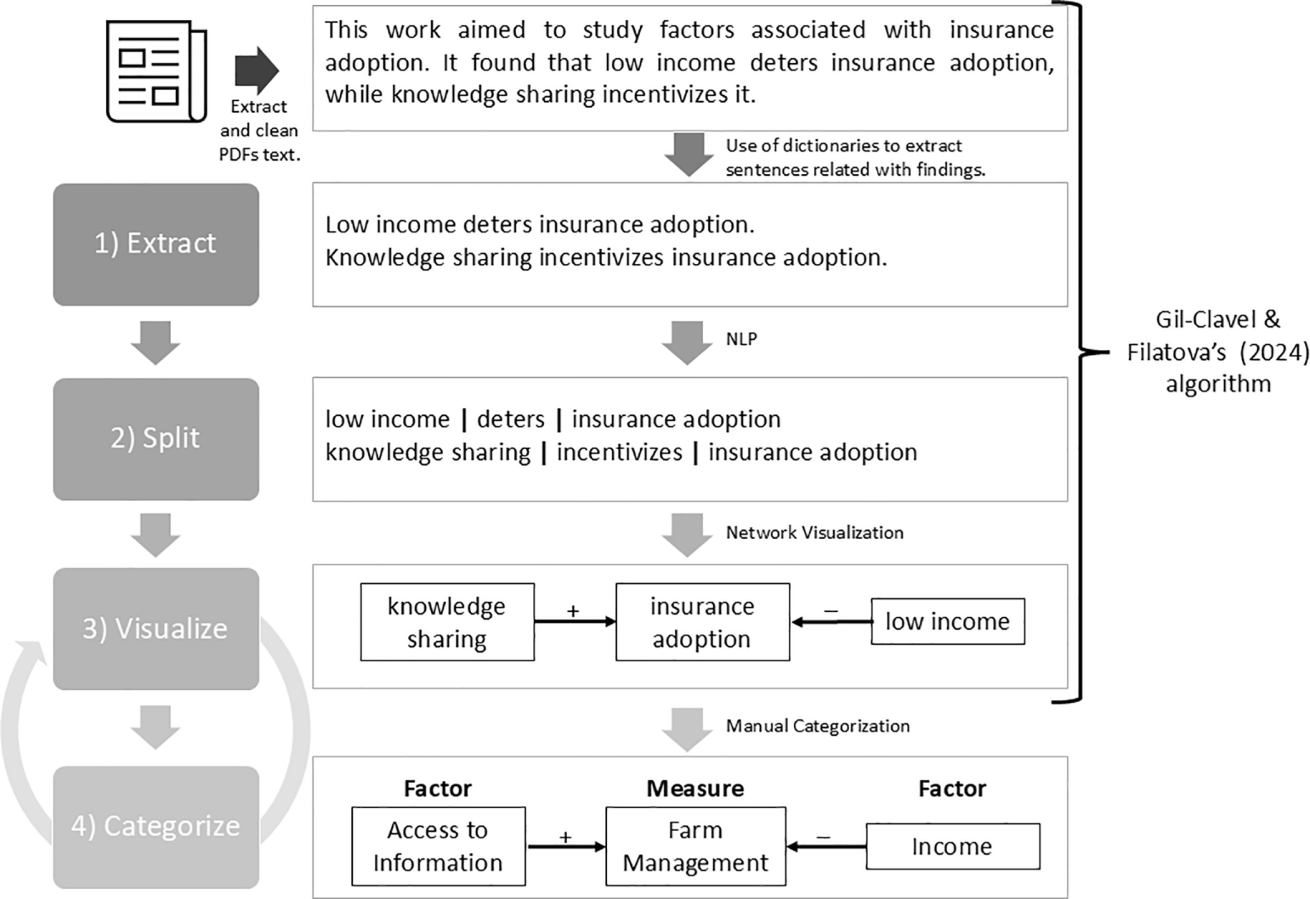
Workflow and the NLP-enhanced algorithm. The algorithm extracts relationships between the reported adaptation measures and their associated factors from the articles’ findings.

Fig 1 shows a visual summary of Gil-Clavel & Filatova’s [34] algorithm. First, once the text from the PDFs is extracted and cleaned, the algorithm identifies the Abstract, Discussion, and Conclusions sections of each article and extracts sentences reporting findings. For this, we labeled around 4000 sentences as either “Finding” or not and then trained a spaCy text categorization model (spaCy, n.d.-b). This is done by splitting the labeled data into the training (75%) and validation (25%) sets. Based on this configuration, we got an 80% categorization score. This is considered good as the model is 30 percentual points better than random chance, and it does not overfit the data. Second, using NLP, specifically Parts-Of-Speech (POS), our algorithm splits the Findings-sentences into subject, verb, and object. We use the python package Spacy [35] together with the ScispaCy model “en_core_sci_lg” for processing biomedical, scientific, or clinical text [36]. We chose ScispaCy because it has a 97% POS sentences segmentation accuracy [36]. This means that the model is very good at finding the POS of biomedical, scientific, or clinical text. However, to make the ScispaCy model more accurate to our specific usage, we utilized Prodigy [37] to update the Name-Entity recognition rules. Prodigy is a software to create, train, and evaluate data for ML models [37]. Hence, we manually coded the names and entities that could be considered as CCA measures and factors. Once the ‘en_core_sci_lg’ model is updated, we modify an algorithm that uses POS to return all the different subjects, verbs, and objects in a sentence. Third, based on the type of association a verb denotes, we replace verbs with their associated signs ( + positive; - negative; + /- neutral). For example, “increase,” “prevent,” and “relate” would be translated into positive “+,” negative “-,” and neutral “+/-” associations, respectively. In this sense, a positive association between a factor and a measure could be interpreted as the factor likely driving CCA. We reframe from claiming identified causalities, since social scientists typically identify correlation rather than causation [38]. Using this information – subject, sign, and object – we visualize our Findings as a directed network, where the nodes are the subjects and objects, and the links denote signs according to the most frequently mentioned association. As a fourth step, we categorized the nodes in the network based on whether they denote a CCA measure or factor. The final lists of measures and factors are in S1 Appendix and S4 Appendix, respectively. S3 Appendix shows an example of the algorithm applied to fewer words.

After categorizing all terms, we derive a database by saving the CCA measures and their factors reported in the articles as new columns [39]. We save the CCA measures as a categorical variable and the type of adaptation as a dichotomous variable, equal to 1 (0) in the case of Transformational (Incremental) CCA. We store all factors associated with CCA as dichotomous, indicating if they are discussed in the Findings (1) or not (0). Consequently, for each CCA measure discussed in the Findings, we collect the values of the factors considered influential for farmers’ CCA by the authors of reviewed papers. The results reported here are based on the dataset consisting of 1013 rows and 25 columns. There are more rows than articles (N = 281) because some articles cover multiple regions and CCA types, resulting in repeated entries. Lastly, the columns also contain the articles’ metadata, such as an article ID [39].

### Differences in the factors between incremental and transformational adaptation

To elicit the differences between incremental and transformational adaptations, we run a logit model (Equation 1).


Transformational~Factors+Region+Article Id. (1)

In Equation 1, the outcome variable is the type of adaptation, which is dichotomous, indicating the adaptation measure discussed in the article Findings: Transformational (1) or Incremental (0). We collect relevant Factors for each type of CCA (Incremental vs. Transformational) by iterating over the identified nodes across all networks (Steps 3-4, Fig 1), and classify them according to the six categories (S1 Appendix). We also add the categorical variable *Region*: Africa, Asia, Europe (reference category), Latin America and the Caribbean (LAC), Northern America, and Oceania. Lastly, we also control (in the statistical terms) for Article ID because there are some articles that cover more than one region and that talk about both transformational and incremental adaptation.

### Patterns in factors found in different regions of the world

To uncover potential patterns in the mechanisms found in different world regions, we run a multinomial model (Equation 2).


Region~Transformational+Factors+Article Id. (2)

In Equation 2 the outcome variable is the variable Region. As before, Region is a categorical variable with the values Africa, Asia, Europe (reference category), Latin America and the Caribbean (LAC), Northern America, and Oceania. The independent variables Factors and Transformational are already explained in Equation 1.

## Results

Our data was retrieved from Scopus [25] during the first week of August 2022, containing articles published up to August 2022. We use Scopus because it has better coverage than Web of Science [40]. This allows us to build a representative sample of research articles published in English around the world. The articles were retrieved from Scopus in an inclusive manner, meaning that initially we did not constrain our search to only farmers. Instead, we downloaded all articles about human CCA (S2 Appendix contains all the search words), which belonged to the categories: Multidisciplinary, Social Sciences, Arts and Humanities, and Environmental Science.

To retrieve the articles, we performed similar steps as those followed by Berrang-Ford et al. [27] and Thomas et al. [16]. First, we searched for articles related to CCA using the search terms “climate change,” “social change,” and “regime shift” (see S2 Appendix). This resulted in 30,000 unique articles. As not all the articles were about the studied topic (factors associated with CCA measures), we needed to categorize the articles as “relevant” or “irrelevant.” For this, we organized a labeling session using the expertise of eight PhD candidates specializing in CCA. Second, to simplify the labelers work, we performed a Non-Negative Matrix Factorization on the articles’ titles and abstracts and then gave each labeler only the articles corresponding to one cluster. This facilitated labeling the articles as relevant or irrelevant for the study of CCA factors. Third, using the articles clustered in that way, we used ASReview [41] to manually categorize 5% of the articles as relevant or irrelevant to the study of CCA based on their abstracts. To train the participants, we explained the problem to them, showing several examples of the type of articles’ abstracts that were useful to our study. In terms of the active learning classifier, we used the following settings: feature extraction technique – TF-IDF; classifier – Naïve Bayes; query strategy – Mixed (95% Maximum and 5% Random); and balance strategy – Dynamic resampling (Double).

The labeling session lasted 4 hours, at the end of which we had 1600 labeled articles with 33% identified as relevant. Fourth, to classify the rest of the articles, we trained a simple perceptron classifier with a Binary Cross Entropy and Sigmoid loss function (BCEWithLogitsLoss function from the python package PyTorch [42]). The model had 79% accuracy and resulted in 2438 articles classified as related to human adaptation to climate change. Fifth, we validated that the articles were about the factors associated with human CCA by checking all the abstracts from the articles. This resulted in around 700 articles. Finally, from the 700 articles, we kept only those that were related to farmers’ CCA, in other words we looked for those that contained the words “farmer” and “agriculture” in either title or abstract. Our final database consists of around 281 articles published between 2005 and 2022.

### Descriptive statistics

**Fig 2:**
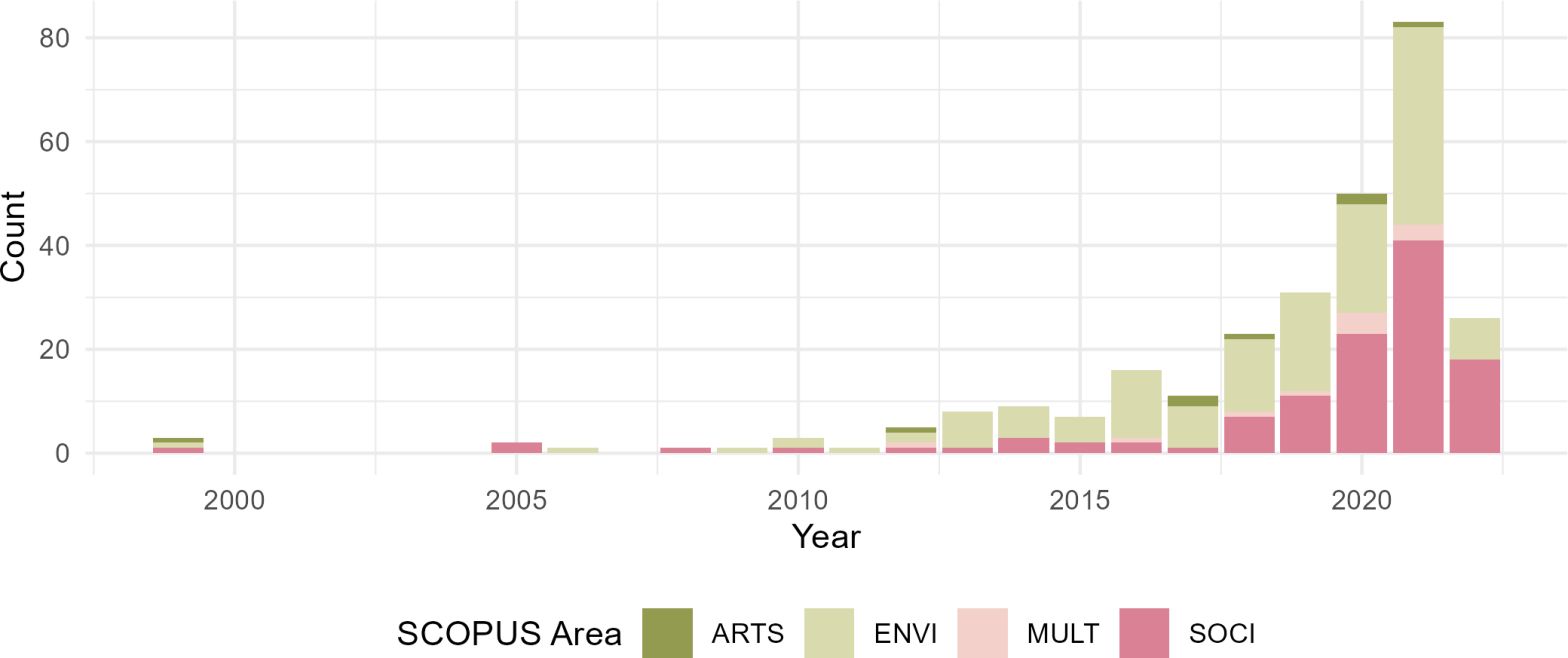
Distribution of articles by year and Scopus subject area (N = 281). The acronyms stand for: Arts and Humanities (ARTS); Environmental Science (ENVI); Multidisciplinary (MULT); and Social Sciences (SOCI).

Fig 2 shows the distribution of articles by year and Scopus subject area [43]. The acronyms stand for: Arts and Humanities (ARTS); Environmental Science (ENVI); Social Sciences (SOCI); and Multidisciplinary (MULT). As Fig 2 shows, our database only contains 13 papers about farmers’ CCA that were published before 2011. After 2010, the number of articles about farmers’ CCA started to increase exponentially, reflecting the typical trend of a research topic evolution [44] and the objectively increasing attention to adaptation due to exacerbating climate-induced hazards.

We checked the method employed in the articles by analyzing whether the title, abstract, or methodology reported any qualitative or quantitative vocabulary. For this, we adapted the database used in Castro Torres & Akbaritabar [45]. We were able to identify vocabulary related to the method type for 82% of the articles. Of the 82%, 36% used a quantitative approach, 22% were qualitative, 32% used mixed methods, and the last 11% were literature reviews.

**Fig 3:**
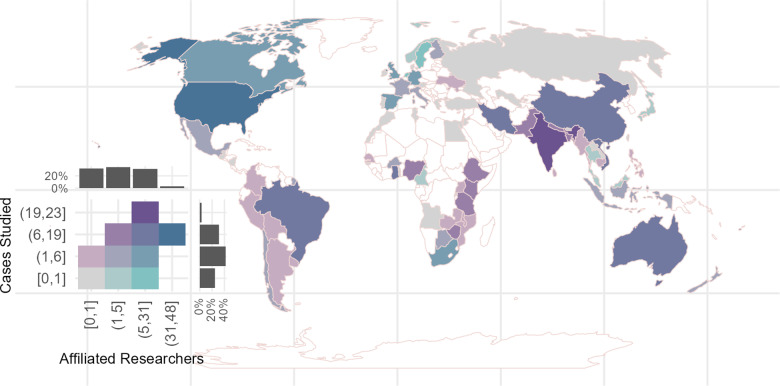
Distribution of articles about farmers’ adaptation to climate change by researchers’ affiliation and cases studied. Categories represent the quantiles [0, 0.33], (0.33, 0.66], (0.66, 0.99], and (0.99, 1]. The histogram represents the aggregate number of countries in each category. Figure made by us with our own data.

Following Nalau & Verrall [46], we plot the distribution of the authors’ affiliations and the distribution of the countries where the research was based (Fig 3). The researchers’ affiliations were derived from the articles’ metadata. The articles’ studied countries were derived from their title, abstract, or conclusions. Of the 281 articles, 80% reported the analyzed city or country. If the article reported the city, we mapped it into its corresponding country. The other 20% of the articles reported the regions where the studies were performed, for example, Sub-Saharan Africa. We did not consider this 20% of articles for Fig 3, but we did include them in the multinomial model analysis. In Fig 3, the countries’ colors are assigned depending on the number of affiliated authors and the number of articles focused on the country, serving as a proxy for the knowledge intensity about CCA in each region. This is done by assigning these numbers to their corresponding quantiles, with the quantiles’ intervals being [0, 0.33], (0.33, 0.66], (0.66, 0.99], (0.99, 1]. In the intervals [0, 0.33] and (0.33, 0.66] the “]” means that 0.33 is part of the first interval, while “(” means that 0.33 is not considered in the second interval. However, for clarity, we show the numbers to which the quantiles correspond.

Many of the articles study African and Latin American countries (Fig 3, see the 0.33 to 0.99 quantiles), but the number of researchers affiliated with those universities is in the lowest quantile interval, 0 to 0.33. In the cases of Asia and Oceania, the number of affiliated researchers and cases studied seems quite balanced, meaning that both quantiles belong to the interval 0.66 to 0.99. For the European countries, there is little research done about those countries (Fig 3, see the lowest quantile), but many researchers are affiliated to those universities (Fig 3, quantile 0.33 and 0.99). Finally, for the North American countries, we see that the number of researchers affiliated with Canadian universities belongs to the 0.66 to 0.99 quantile, while the number of articles studying Canada is in the 0.33 to 0.66 quantile. United States of America could be considered an outlier, as its number of affiliated researchers –48– and cases analyzed –23– are the biggest in the database, which is also what Nalau & Verrall [46] reported.

**Fig 4:**
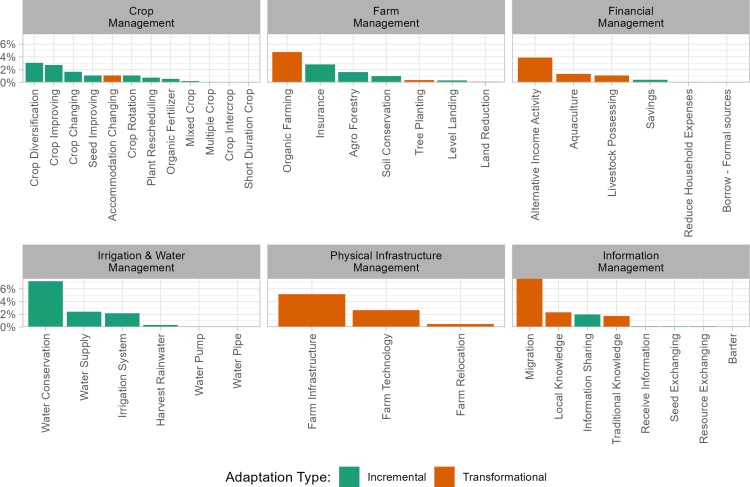
Bar charts of the percentage of times the adaptation options were mentioned in the articles broken down by Adaptation Category (horizontal axis) and Adaptation Type (Incremental vs. Transformational). The values add up to 65%; the remainder corresponds to the broad terms ‘climate change adaptation’ and ‘climate change transformation adaptation’.

Farmers’ CCA can take various forms and may be referred to by different terms, especially when studied through the lens of different disciplines. To account for this, we used a range of generic search terms, like ‘adaptation’, together with the expressions elicited from the Findings’ (Steps 3 and 4, Fig 1) to expand the vocabulary of the possible relevant terms describing climate adaptation measures. We then classify the extensive list of terminology describing farmers’ CCA according to the six categories (see (i)-(vi) in section ‘Categories of farmers’ climate change adaptation measures’, and the full list in S1 Appendix). Fig 4 shows the percentage of articles that refer to the adaptation measures. In 32% of the articles, researchers did not mention the specific adaptation measure undergone; instead, researchers referred to broad terms such as “climate change adaptation” or “transformational adaptation.”

### Factors associated with farmers’ climate change adaptation

**Fig 5:**
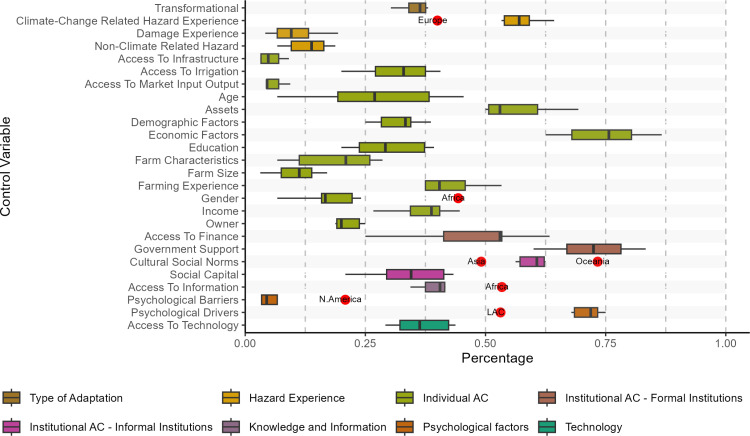
Boxplots of the percentage of times the transformational adaptation and the factors appear in the articles’ Findings by region. For each of the Factors red dots represent outliers labeled with the Regions they represent. Namely, if we detect an outlier (i.e., values below/above the expected variation) in a specific Region for a specific Factor, then a red dot is reported left/right of the boxplot. A boxplot represents the minimum, maximum, and the interquartile range in each Factor category. AC stands for Adaptive Capacity.

By analyzing the clusters generated using this algorithm [33], we identify 25 categories of Factors (Table D1 in S4 Appendix shows the complete list of factors by category and their final network, respectively). Fig 5 shows the boxplots of the percentage of times each Factor is discussed in the articles by region (Table D2 in S4 Appendix shows the percentages). Based on the boxplots, we can see that there are some outliers. For example, in the case of the Factor ‘Climate-Change Related Hazard Experience’, Europe has a value that is below the expected variation. This means that in comparison with articles that discuss other regions, articles that discuss European countries do not tend to connect Climate-Change Related Hazard Experience with undertaking CCA measures. As a region, Africa has percentages that are above the expected variation for Gender and Access to Information. This, similar to Europe, means that articles about African countries tend to link Gender and Access to Information more often with CCA measures compared to articles about other countries.

**Fig 6:**
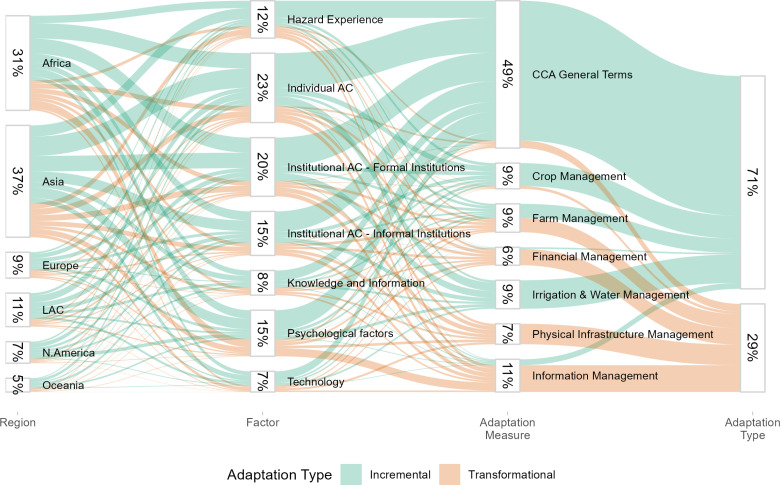
Distribution of reported Findings across Regions, adaptation Factors, and climate change adaptation measures. String colors represent adaptation type (Incremental vs. Transformational). AC stands for Adaptive Capacity.

Notably, the patterns of reported adaptation Factors (using the Overall Groups presented in Table D2 in S4 Appendix), and adaptation measures by type of adaptation (Incremental and Transformational) are unevenly distributed across regions (Fig 6). Specifically, 71% of the CCA measures reported in the articles are Incremental, while 29% are Transformational. In terms of the regions, Asia was found to be the most studied region and Oceania the least, with 37% and 5% articles analyzing these continents, respectively. Individual adaptive capacity (Individual-AC in Fig 6) is the most and Technology the least reported Factors, namely 23% and 7%, respectively. Finally, 49% of the Findings mention CCA measures in general terms hindering us from attributing these findings to any of the specific CCA measures groups. Still, with respect to the specific CCA measures, the biggest percentage (11%) of the Findings across the reviewed articles reported Information Management as the farmers’ CCA measure, and only 6% reported Financial Management.

### Differences in the factors between incremental and transformational adaptation

**Fig 7:**
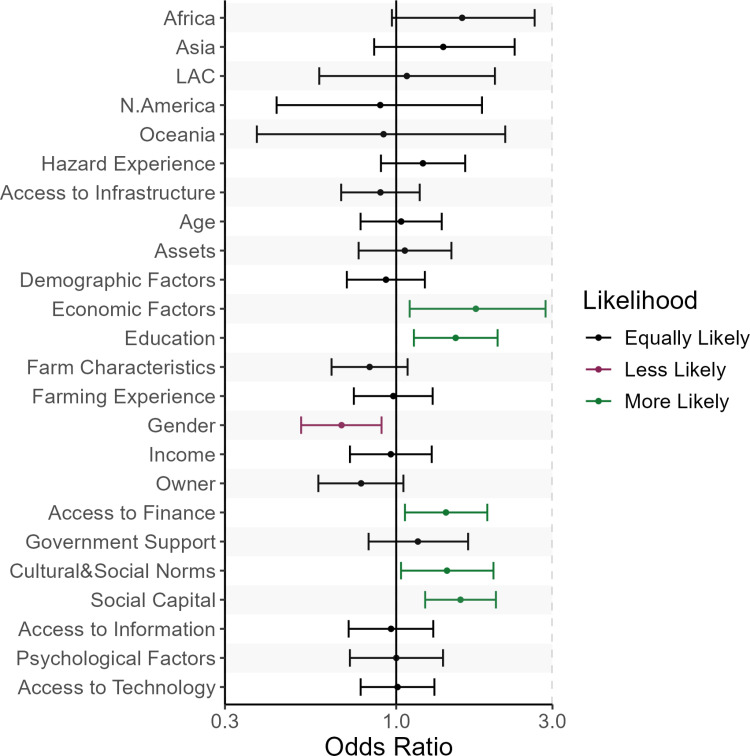
The likelihood of Factors being associated with Transformational compared to Incremental climate change adaptation: (>1 - green) more likely, (<1 - red) less likely, (=1 - black) equally likely. Here we report the dot-plot of the odd ratios of the logit model (Eq. 1) where the outcome variable is Incremental (0) vs. Transformational (1). The coefficients include their 95% Confidence Interval.

Diving further, we explore which Factors are reported to be associated with Transformational CCA more likely than with Incremental (Fig 7). We visualize our estimates using the dot-plot of the logit model (Eq. 1) coefficients with their 95% confidence intervals. The reference category is the Findings in articles about European countries that do not find any of the Factors to be associated with Transformational adaptation. As an example of the interpretation of the coefficients, let us focus on Economic Factors: compared to articles about Incremental adaptation, articles about Transformational adaptation tend to report Economic Factors more often as factors associated with farmers’ CCA.

Based on the results in Fig 7, no statistically significant differences are/were found in the number of times articles about different regions report Transformational adaptation measures in their Findings. This result allows us to interpret the rest of the variables without considering the region as something that could modify the direction of the association. All factors that are reported to be more likely associated with farmers’ transformational CCA rather than incremental CCA (green bars, Fig 7) characterize various aspects of adaptation capacity [47]. For example, ‘Economic Factors’ and ‘Education’ constitute individual adaptive capacity, while ‘Access to Finance’ characterizes the adaptive capacity of formal institutions. In turn, Factors like ‘Cultural and Social Norms’ and ‘Social Capital’ signal the adaptive capacity of informal institutions. Notably, Gender is the only variable less likely to be mentioned as a factor in transformational adaptation compared to incremental adaptation (red bar, Fig 7). All other factors are equally likely to appear in articles that discuss incremental compared to transformational CCA of farmers.

### Patterns in adaptation factors across different world regions

**Fig 8:**
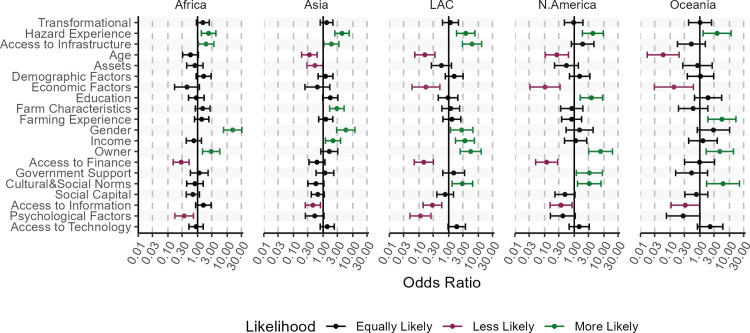
The likelihood of Factors being associated with farmers’ climate change adaptation in world regions compared to Europe as the reference category: (>1 - green) more likely, (<1 - red) less likely, (=1 - black) equally likely. Here, we report the dot-plot of the odd ratios from the regional multinomial model (Eq. 2) with 95% Confidence Intervals.

Climate impacts and adaptation is likely to be uneven across world regions. To get insights into what empirical literature reports here, we assess which Factors are likely to be associated with farmers’ CCA worldwide (Fig 8). As the reference category for each factor, we use articles that study incremental adaptation in European countries and find no associations between this factor and farmers’ CCA. We illustrate the regional differences using the dot-plot of the odd ratios of the multinomial model (Eq. 2) with their 95% confidence interval. To illustrate the interpretation of coefficients, consider the Factor ‘Gender’ for Asia (Fig 8). In this case, the coefficient indicates that compared to articles reporting farmers CCA in European countries, those about Asian countries are more likely to report Gender as the factor associated with farmers’ CCA.

Regarding adaptation type, articles studying farmers’ CCA in other world regions report Transformational CCA (instead of Incremental, Fig 8) as frequently as do articles about European farmers. Among Factors associated with farmers’ CCA, ‘Hazard Experience’ appears more frequently in all regions compared to Europe. For Factors denoting individual adaptive capacity – from ‘Access to Infrastructure’ to ‘Owner’ (Fig 8) – we find differences between high-income and low/middle-income countries. Namely, compared to European countries, Factors ‘Access to Infrastructure’, ‘Gender’, and ‘Income’ are more frequently reported to be associated with farmers’ CCA in Africa, Asia, and LAC. Regions N. America and Oceania show no statistical differences from Europe.

Besides individual adaptive capacity, institutional adaptive capacity, including formal and informal institutions, plays a role. Among formal institutions, we find that ‘Access to Finance’ appears less often as a factor associated with farmers’ CCA in articles about African, LAC, and N. American countries compared to articles about European countries. Factor ‘Government Support’ is more likely to be associated with farmers’ CCA in articles about N. American countries, while its likelihood appears similar across other regions. Moving to the Informal Institutions, Fig 8 shows that Factors related to ‘Cultural and Social Norms’ are more likely to be mentioned in articles about LAC, N. America, and Oceania than about Europe. In the case of ‘Social Capital’, it is statistically equally likely to be mentioned for all regions.

Finally, we explore Factors ‘Access to Information’, ‘Psychological Factors’, and ‘Access to Technology’ belonging to the umbrella categories ‘Knowledge and Information’, ‘Psychological Factors’, and ‘Technology’, respectively (S4 Appendix). Fig 8 shows that ‘Access to Information’ is less likely (in Asia, LAC, N. America and Oceania) or equally likely (in Africa) to be associated with farmers’ CCA, compared to research reporting about European farmers. ‘Psychological Factors’ are, on average, less likely to be associated with farmers’ CCA in all regions compared to European countries. Finally, the Factor ‘Access to Technology’ is equally likely to be associated with farmers’ adaptation across all regions.

## Discussion and conclusions

While evidence of factors affecting CCA decisions is mounting, systematizing this information across quantitative and qualitative strands of literature is challenging. This article contributes to the literature in two ways. First, we proposed a novel way to study articles from different disciplines regardless of whether they use qualitative, quantitative, or mixed methods. Our methodology relies on using NLP to derive a database of interlinked concepts describing factors and metadata, which was then used as input for multinomial models. Second, we used this methodology to perform a comparative analysis of farmers’ factors for CCA using generalized linear models. Specifically, we study whether factors associated with farmers’ adaptation vary depending on the type of adaptation (incremental vs. transformational) and whether there are differences in the adaptation factors in different regions of the world. Our findings show that 30% of the articles analyzed farmers’ Transformational adaptation, with no significant differences revealed across regions.

Differentiating among types of adaptation – Incremental vs. Transformational – we find that factors related to adaptive capacity are more likely to be associated with farmers’ Transformational CCA. These include individual adaptive capacity (‘Economic Factors’ and ‘Age’), as well as those related to formal (‘Access to Finance’) and informal (‘Cultural and Social Norms’ and ‘Social Capital’) institutions. These results reflect the difficulties in implementing Transformational CCA that are related to large initial costs [9,29] and other adaptation constraints beyond just economic and financial [16]. ‘Economic Factors’ and ‘Access to Finance’ could help overcome these barriers, offering policy levers to enable Transformational CCA. Notably, other factors – ‘Hazard Experience’, ‘Governmental support’, or ‘Access to information’ – appear equally important for Incremental and Transformational CCA. This finding aligns with the discourse in the theoretical literature that points to a grey zone between transformational and incremental CCA [9,48]. Notably, ‘Gender’ is the only variable that is less likely to be mentioned as associated with farmers’ Transformational adaptation compared to articles that discuss Incremental adaptation. This might be because female farmers often have less access to finance [49] and other resources commonly associated with Transformational CCA. Therefore, gender-specific subsidies could support women also to pursue transformational CCA.

In terms of whether there are differences in farmers’ adaptation factors in different regions of the world, our results show that factors vary by region. ‘Gender’ is a factor that tends to be more frequently reported in low- and middle-income countries, possibly because in those regions agrifood systems are a more important source of livelihood for women than for men compared to high-income regions [50]. Also, ‘Income’ and ‘Access to Infrastructure’ are more frequently mentioned in the article findings on low- and middle-income countries, compared to high-income countries. This could point to uneven starting conditions for farmers across world regions where not all African, Asian, and LAC farmers have Access to Infrastructure, in contrast to most European, North American, and Oceanian farmers [16]. Identifying such differences in adaptation factors could help prioritize the type of CCA support that Global South might benefit most, and to streamline the global negotiations on loss and damage and Global Adaptation Fund at the annual Conference of Parties.

Our generalizable and reproducible approach presents a novel way to process and synthesize large-scale literature findings, moving beyond merely summarizing abstracts and counting key terms. This is increasingly necessary as the amount of literature grows exponentially [44], including in the CCA domain [46]. Specifically, we combine methodologies stemming from machine learning and NLP with statistical analyses to summarize the articles’ findings regarding the CCA measures and factors. This approach helps automize the derivation of databases stemming from peer-reviewed articles, which so far has been done manually, requiring many hours of intensive labor [51].

While this article made a step forward, it is not without limitations to be addressed by future research. First, our methodology depends on manually creating dictionaries to classify the measures and factors in the findings, in order to create a more transparent and interpretable process [34]. These dictionaries can be incomplete, as they might omit some farmers’ CCA measures or might have missed some discipline-specific terms referring to the same factors. Second, depending on the research approach, some of the farmers’ CCA measures could be classified into multiple categories at the same time. For example, migration is considered a Social Activity by Shaffril et al. [12], while it is considered Income Diversification by Bahinipati et al. [10]. Furthermore, the classification of incremental and transformational measures used was based on the Asian context [12]. Farming practices worldwide differ significantly from the reason to farm (subsistence-based to big-scale, profit-based), the equipment available, or the climatic conditions. Therefore, whether something is considered transformational or not is highly dependent on the context; the definitions used here might not be applicable universally. Additionally, the line between transformational and incremental CCA is not clear-cut [9,32]. Thus, the classification of transformational versus incremental operationalized in this article is not universal. However, we believe that our results are still representative of the broad categories to which the farmers’ CCA measures belong (S1 Appendix).

Despite the limitations, speeding-up the analysis of literature lays a foundation for climate researchers and policy makers. As our proposed methodology provides them with the required insights into which factors play a role in a region. These insights can then help policy makers support farmers in their journey to incrementally or transformational adapt to climate change.

## Supporting information

S1 Appendix Farmers’ adaptation measures dictionary of terms by type of adaptation. (DOCX)

S2 Appendix Search terms. (DOCX)

S3 Appendix Algorithm applied to find factors associated with farmers’ climate change adaptation. (DOCX)

S4 Appendix Tables of farmers’ adaptation factors. (DOCX)
