# Supplementary material for: Incremental and transformational climate change adaptation factors in agriculture worldwide: A comparative analysis using natural language processing
Source: PLoS One. 2025 Mar 19;20(3):e0318784. doi: 10.1371/journal.pone.0318784 (PMC11922273; doi:10.1371/journal.pone.0318784)
Supplement: S1 Appendix — (DOCX) [file pone.0318784.s001.docx]

# **Supporting Information**

This file contains all the supporting information of the article “Incremental and Transformational Climate Change Adaptation Factors in Agriculture Worldwide: A Comparative Analysis using Natural Language Processing” by Sofia Gil-Clavel, Thorid Wagenblast, and Tatiana Filatova.

## **Appendix A: Farmers’ Adaptation Measures Dictionary of Terms by Type of Adaptation**

| **Incremental adaptation** | **Transformational adaptation** |
| --- | --- |
| **I. Crop Management** | |
| Crop Changing | Accommodation Changing |
| Crop Diversification | Farm Infrastructure |
| Crop Improving |  |
| Crop Intercrop |  |
| Crop Rotation |  |
| Mixed Crop |  |
| Multiple Crop |  |
| Organic Fertilizer |  |
| Organic Insecticide |  |
| Organic Pesticide |  |
| Organic Reutilizer |  |
| Plant Rescheduling |  |
| Seed Improving |  |
| Short Duration Crop |  |
| **II. Irrigation and Water Management** | |
| Harvest Rainwater |  |
| Irrigation System |  |
| Water Conservation |  |
| Water Pipe |  |
| Water Pump |  |
| Water Supply |  |
| **II. Farm Management** | |
| Agro Forestry | Dry Farming |
| Homestead Gardening | Land Reduction |
| Indoor Farming | Orchard Farming |
| Insurance | Organic Farming |
| Level Landing | Tree Planting |
| Organic Farming |  |
| Soil Conservation |  |
| **IV. Financial Management** | |
| Borrowing from formal sources | Alternative Income Activity |
| Borrowing from informal sources | Aquaculture |
| Loan Taking | Livestock Possessing |
| Reduce Household Expenses |  |
| Savings |  |
| **V. Physical Infrastructure Management** | |
| Farm Infrastructure | Farm Infrastructure |
|  | Farm Relocation |
|  | Farm Technology |
| **VI. Information Management** | |
| Barter | Local Knowledge |
| Food Exchanging | Migration |
| Information Sharing | Traditional Knowledge |
| Receive Information |  |
| Resource Exchanging |  |
| Seed Exchanging |  |
| Barter |  |
