# Supplementary material for: Incremental and transformational climate change adaptation factors in agriculture worldwide: A comparative analysis using natural language processing
Source: PLoS One. 2025 Mar 19;20(3):e0318784. doi: 10.1371/journal.pone.0318784 (PMC11922273; doi:10.1371/journal.pone.0318784)
Supplement: S2 Appendix — (DOCX) [file pone.0318784.s002.docx]

# **Supporting Information**

This file contains all the supporting information of the article “Incremental and Transformational Climate Change Adaptation Factors in Agriculture Worldwide: A Comparative Analysis using Natural Language Processing” by Sofia Gil-Clavel, Thorid Wagenblast, and Tatiana Filatova.

## **Appendix B: Search terms**

The search terms used to retrieve the articles’ abstracts and metadata are:

**1^st^ layer: Basic terms for the phenomena itself (social transformation)**

- ‘social change'
- ‘structural change’ AND [society OR social]
- transition* (could also be with -s, -al) AND [society OR social]
- transformation* (could also be with -s, -al) AND [society OR social]
- transformative AND [society OR social]
- revolution* AND [society OR social]
- ‘regime shift’ AND [society OR social]
- ‘tipping point’ AND [society OR social]
- ‘tipping element*AND [society OR social]
- ‘critical transition’ AND [society OR social]
- threshold AND [social OR econom* OR behavio* OR policy OR political]
- ‘state shift’
- cascad*
- domino
- ‘chain reaction’
- ‘relocation’ OR ‘retreat’ OR ‘buyout*’ or ‘ buy out’

**2^nd^ layer (use #1 with either 2.a or 2.b):**

1. terms specific to our hazard of interest: ‘flood*’, deluge, storm, hurricane, typhoon, heavy rainfall, dike, levee, climate OR ‘climate change’
2. Terms for other hazards that can also be of interest: earthquake, landslide, wildfire, drought

**3^rd^ layer (use first 3.a with #2.a, if too many apply also #1; then use 3.b with #1; then use 3.a with 3.b):**

1. terms specific to a SSH domain: the below disciplines need to be used separately in the searchers
2. Governance/Public Administration: ‘

- punctuated equilibrium’
- ‘multiple streams’
- ‘advocacy coalition*’
- ‘ecology of games’

1. case-specific terms (the cases of past transformative responses to hazards I know of):

- ‘Room for the River’ OR ‘Room for River’ OR ‘Space for River’
- Deltaworks or Deltawerken
- Afsluitdijk
- Houston OR [Houston AND Galveston]
