# Supplementary material for: Incremental and transformational climate change adaptation factors in agriculture worldwide: A comparative analysis using natural language processing
Source: PLoS One. 2025 Mar 19;20(3):e0318784. doi: 10.1371/journal.pone.0318784 (PMC11922273; doi:10.1371/journal.pone.0318784)
Supplement: S3 Appendix — (DOCX) [file pone.0318784.s003.docx]

# **Supporting Information**

This file contains all the supporting information of the article “Incremental and Transformational Climate Change Adaptation Factors in Agriculture Worldwide: A Comparative Analysis using Natural Language Processing” by Sofia Gil-Clavel, Thorid Wagenblast, and Tatiana Filatova.

## **Appendix C: Algorithm applied to find factors associated with farmers’ climate change adaptation**

This section shows an example of how we used Gil-Clavel & Filatova’s [1] algorithm to find the factors applied to a subsample of the reviewed articles’ Findings. The subsample consists of all the Findings reporting climate change adaptation associations. For this, we used the following regular expressions: ‘adaptat[a-z]* [a-z]*(\s|) climate change’, ‘adaptat[a-z]* [a-z]* measure’, ‘adaptat[a-z]* [a-z]* capacity’, ‘farm[a-z]* [a-z]* adaptat[a-z]*’, ‘climate adaptat[a-z]*’, ‘adaptat[a-z]*[a-z]* measure’. Fig 9 shows the clustered network.

In Fig 9, the color of the nodes and the central terms denote the cluster to which the variables belong to, for example, the biggest cluster concerns “climate adaptation”. Then, based on Gil-Clavel & Filatova’s [1] algorithm, the closer the terms are to the center the more frequently the words appear in the articles. Finally, the links between the terms show whether the terms are connected, the links’ thickness denotes the frequency, and their color denotes the more frequent association between the terms (i.e., green – positively associated, yellow – neutrally associated, and red – negatively associated). Therefore, we can interpret Fig 9 in the following manner. Food security, place attachment, and local development are frequently reported to have a positive association with climate adaptation. Culture and industry are frequently reported to have a negative association.


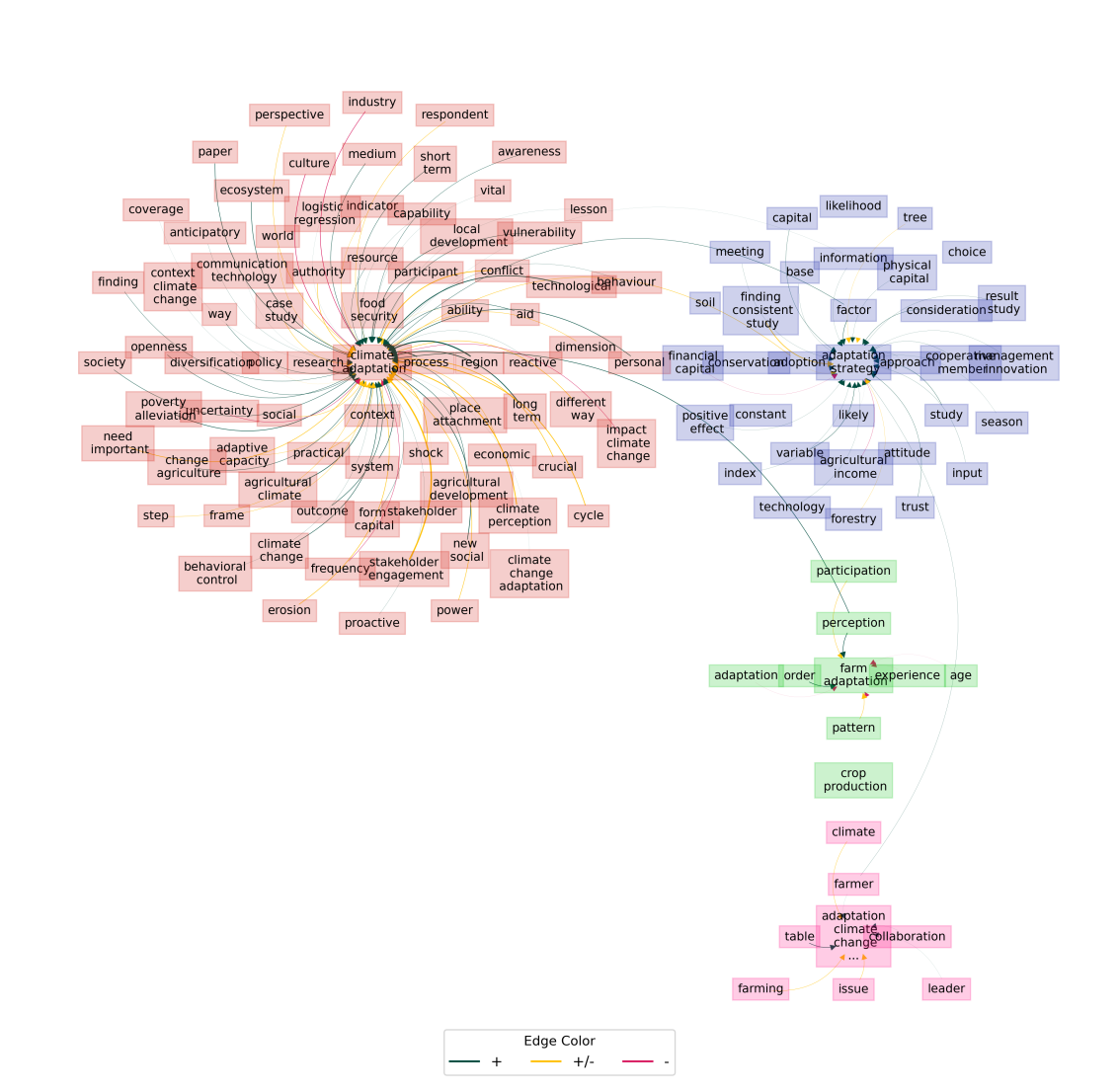


**Fig C1. Clustered networks of nodes targeting nodes containing the word “adaptation.”**

Based on the network, we can identify those terms that can be considered as farmers’ climate change adaptation factors. For this, we relied on our knowledge and on climate change adaptation literature [2,3]. Once this is done, we can cluster those terms under different categories, such as Access to Information and Government Support (Fig 10).


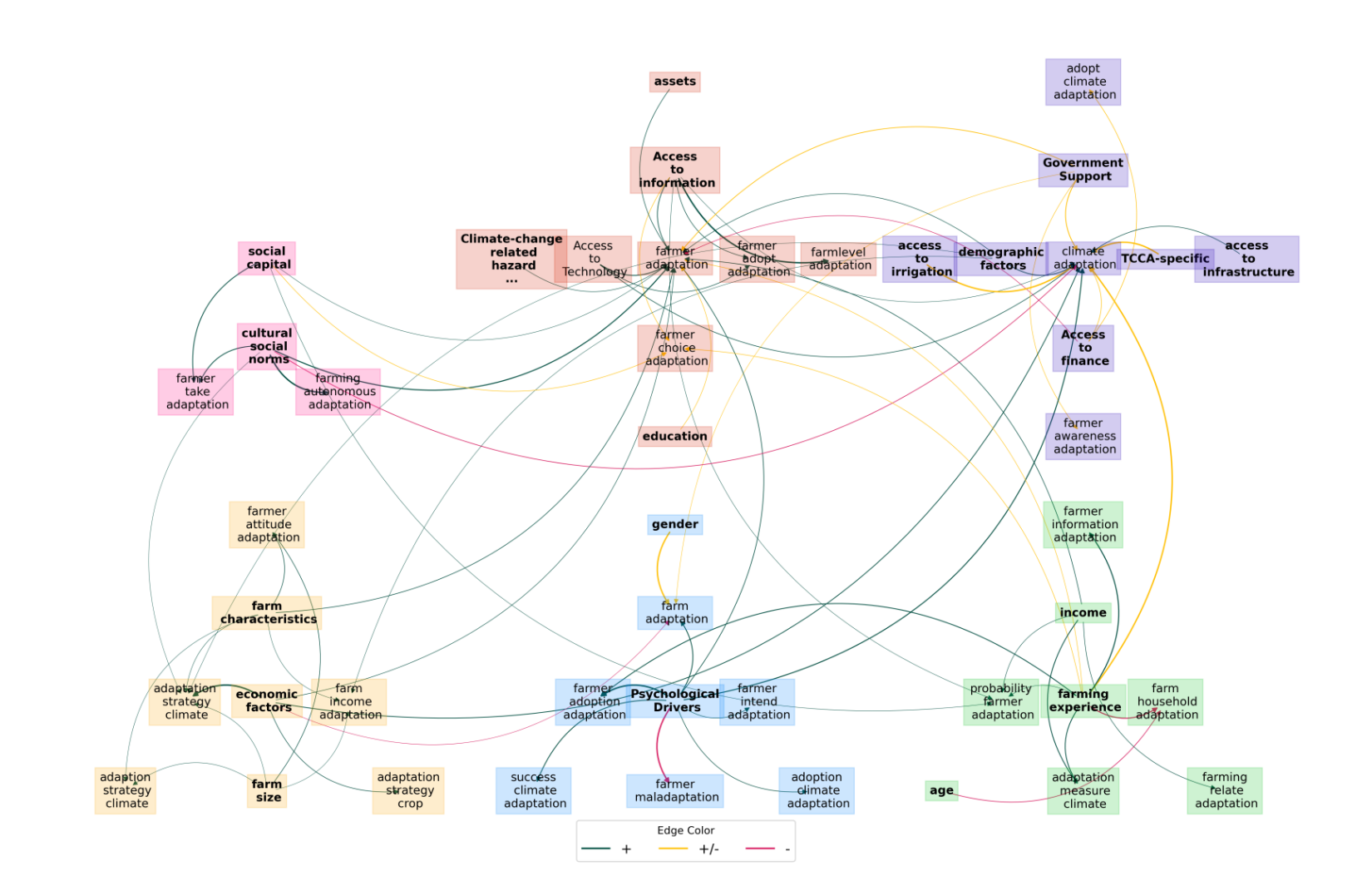


**Fig C2. Clustered networks of nodes targeting nodes containing the word “adaptation” after labeling them with the umbrella categories (in bold).**

## References

1. Gil-Clavel S, Filatova T. Using Natural Language Processing and Networks to Automate Structured Literature Reviews: An Application to Farmers Climate Change Adaptation [Internet]. arXiv; 2024 [cited 2024 Oct 4]. Available from: https://arxiv.org/abs/2306.09737v2

2. Berrang-Ford L, Siders AR, Lesnikowski A, Fischer AP, Callaghan MW, Haddaway NR, et al. A systematic global stocktake of evidence on human adaptation to climate change. Nat Clim Change. 2021 Nov;11(11):989–1000.

3. van Valkengoed AM, Steg L. Meta-analyses of factors motivating climate change adaptation behaviour. Nat Clim Change. 2019 Feb;9(2):158–63.
