# Supplementary material for: Incremental and transformational climate change adaptation factors in agriculture worldwide: A comparative analysis using natural language processing
Source: PLoS One. 2025 Mar 19;20(3):e0318784. doi: 10.1371/journal.pone.0318784 (PMC11922273; doi:10.1371/journal.pone.0318784)
Supplement: S4 Appendix — (DOCX) [file pone.0318784.s004.docx]

# **Supporting Information**

This file contains all the supporting information of the article “Incremental and Transformational Climate Change Adaptation Factors in Agriculture Worldwide: A Comparative Analysis using Natural Language Processing” by Sofia Gil-Clavel, Thorid Wagenblast, and Tatiana Filatova.

## **Appendix D: Dictionary of farmers’ adaptation factors**

**Table D1**: Words classified as factors associated with farmers’ climate change adaptation

| **Access To Finance** |
| --- |
| (?:^\|\W)bank(s\|) |
| (?:^\|\W)debt(s\|) |
| (?:^\|\W)invest[a-z]* |
| (?:^\|\W)loan(s\|) |
| (?:^\|\W)payback |
| (?:^\|\W)subsid[a-z]* |
| acces[a-z]* [[a-z]*\s{0,1}]{0,1}credit(s\|) |
| develop[a-z]* [[a-z]*\s{0,1}]{0,1}countr[a-z]* |
| farm[a-z]* [[a-z]*\s{0,1}]{0,1}credit[a-z]* |
| financ[a-z]* [[a-z]*\s{0,1}]{0,1}access[a-z]* |
| financial |
| interest rate(s\|) |
| investment[a-z]* |
| micro(\s\|-\|)credit(s\|) |
| pay(\s\|-\|)off |
| private sector |
| remittance(s\|) |
| resource endowment |
| return[a-z]* [[a-z]*\s{0,1}]{0,1}investment(s\|) |
| return[a-z]* [[a-z]*\s{0,1}]{0,1}period(s\|) |
| social benefit(s\|) |
| social welfare |
| **Access To Information** |
| (?:^\|\W)training(s\|) |
| ^information$ |
| ^knowledge$ |
| acces[a-z]* [[a-z]*\s{0,1}]{0,1}information |
| cellular phone |
| climat[a-z]* [[a-z]*\s{0,1}]{0,1}forecast[a-z]* |
| climat[a-z]* [[a-z]*\s{0,1}]{0,1}inform[a-z]* |
| climat[a-z]* [[a-z]*\s{0,1}]{0,1}service[a-z]* |
| extension contact |
| extension officer(s\|) |
| extension provider(s\|) |
| extension service(s\|) |
| extension worker(s\|) |
| external knowledge |
| group [[a-z]*\s{0,1}]{0,1}learn[a-z]* |
| guid[a-z]* [[a-z]*\s{0,1}]{0,1}support[a-z]* |
| guideline |
| historical knowledge |
| knowledge funda[a-z]* |
| new knowledge |
| scientif[a-z]* knowledge |
| weather information |
| **Access To Infrastructure** |
| access[a-z]* [[a-z]*\s{0,1}]{0,1}infrastructure(s\|) |
| energy infrastructure(s\|) |
| farm characteristic[a-z]* |
| farm infrastructure(s\|) |
| farm[a-z]* [[a-z]*\s{0,1}]{0,1}infrastructure(s\|) |
| infrastructure facilit[a-z]* |
| lack [[a-z]*\s{0,1}]{0,1}access |
| number [[a-z]*\s{0,1}]{0,1}worker[a-z]* |
| road network(s\|) |
| solar electric[a-z]* |
| storage facilit[a-z]* |
| supply tool(\|s) |
| **Access To Irrigation** |
| ^precipitation(s\|)$ |
| access[a-z]* [[a-z]*\s{0,1}]{0,1} water |
| access[a-z]* [[a-z]*\s{0,1}]{0,1}irrigation |
| allocati[a-z]* [[a-z]*\s{0,1}]{0,1} water |
| alternative water(\s\|-\|)source(s\|) |
| availab[a-z]* [[a-z]*\s{0,1}]{0,1}water |
| conflict[a-z]* [[a-z]*\s{0,1}]{0,1} water |
| dictribut[a-z]* [[a-z]*\s{0,1}]{0,1} water |
| difficult hydrology |
| distribut[a-z]* [[a-z]*\s{0,1}]{0,1}irrigation |
| glacial meltwater |
| informal[a-z]* [[a-z]*\s{0,1}]{0,1}water |
| insecur[a-z]* [[a-z]*\s{0,1}]{0,1} water |
| irrigat[a-z]* |
| scarc[a-z]* [[a-z]*\s{0,1}]{0,1} water |
| water [[a-z]*\s{0,1}]{0,1}access[a-z]* |
| water [[a-z]*\s{0,1}]{0,1}allocation(s\|) |
| water [[a-z]*\s{0,1}]{0,1}availab[a-z]* |
| water [[a-z]*\s{0,1}]{0,1}conflict(s\|) |
| water distribution |
| water insecurit[a-z]* |
| water right(\|s) |
| water scarcity |
| water stress |
| **Access To Market Input Output** |
| acces[a-z]* [[a-z]*\s{0,1}]{0,1}market |
| cost[a-z]* [[a-z]*\s{0,1}]{0,1}travel[a-z]* |
| distance [[a-z]*\s{0,1}]{0,1}farm[a-z]* |
| distance [[a-z]*\s{0,1}]{0,1}market[a-z]* |
| farm[a-z]* [[a-z]*\s{0,1}]{0,1}distance(s\|) |
| market [[a-z]*\s{0,1}]{0,1}distance(s\|) |
| travel[a-z]* cost[a-z]* |
| **Access To Technology** |
| (?:^\|\W)technolog[a-z]* |
| (?:^\|\W)television |
| access[a-z]* [[a-z]*\s{0,1}]{0,1}technolog[a-z]* |
| communication network(s\|) |
| equipment(s\|) |
| farm[a-z]* [[a-z]*\s{0,1}]{0,1}technolog[a-z]* |
| innovation |
| internet |
| productive capacity |
| technolog[a-z]* [[a-z]*\s{0,1}]{0,1}access[a-z]* |
| technological device |
| **Age** |
| (?:^\|\W)age(?:$\|\W) |
| (?:^\|\W)aging(?:$\|\W) |
| (?:^\|\W)child(?:$\|\W) |
| (?:^\|\W)elderly(?:$\|\W) |
| (?:^\|\W)old[a-z]*(?:$\|\W) |
| (?:^\|\W)young(?:$\|\W) |
| old[a-z]* farmer(s\|) |
| young[a-z]* |
| **Assets** |
| (?:^\|\W)asset(?:$\|\W) |
| belonging(s\|) |
| capital(s\|) |
| goods |
| possesion(s\|) |
| resourc[a-z]* |
| saving(s\|) |
| valuables |
| wealth |
| **Climate-Change Related Hazard Experience** |
| artic ([a-z\s]*)^{0,1}ice[a-z]* los[a-z]* |
| availab[a-z]* summer season |
| change [[a-z]*\s{0,1}]{0,1}biophysical environment |
| climat[a-z]* [[a-z]*\s{0,1}]{0,1}hazard(s\|) |
| climat[a-z]* [[a-z]*\s{0,1}]{0,1}risk |
| climat[a-z]* [[a-z]*\s{0,1}]{0,1}stress[a-z]* |
| climat[a-z]* [[a-z]*\s{0,1}]{0,1}variability |
| climat[a-z]* [[a-z]*\s{0,1}]{0,1}vulnera[a-z]* |
| coastal flood[a-z]* |
| cold(-\|\s\|)spell(s\|) |
| dissemination [[a-z]*\s{0,1}]{0,1}drought[a-z]* |
| drought(s\|) |
| dry(-\|\s\|)spell(s\|) |
| environmental hazard(s\|) |
| extreme [[a-z]*\s{0,1}]{0,1}cold(s\|) |
| extreme [[a-z]*\s{0,1}]{0,1}event(s\|) |
| extreme [[a-z]*\s{0,1}]{0,1}heat |
| extreme [[a-z]*\s{0,1}]{0,1}precipitation(s\|) |
| extreme [[a-z]*\s{0,1}]{0,1}temperature(s\|) |
| flood[a-z]* |
| hazard experience(s\|) |
| heat(\|-\|\s)wave(s\|) |
| heavy rain[a-z]* |
| hurricane(s\|) |
| increas[a-s]* [[a-z]*\s{0,1}]{0,1}precipitation(s\|) |
| increas[a-z]* [[a-z]*\s{0,1}]{0,1}evaporation(s\|) |
| increas[a-z]* [[a-z]*\s{0,1}]{0,1}rate(s\|) |
| land(s\|-\|)slide(s\|) |
| los[a-z]* ([a-z\s]*)^{0,1}artic ([a-z\s]*)^{0,1}ice[a-z]* |
| monsoon variability |
| natural calamit[a-z]* |
| natural disaster(s\|) |
| natural hazard(s\|) |
| ocean[a-z]* acidification |
| precipitation(s\|) variability |
| prone area(s\|) |
| rainfall [[a-z]*\s{0,1}]{0,1}variability |
| ris[a-z]* ocean[a-z]* temperatur[a-z]* |
| ris[a-z]* sea(\s\|-\|)level |
| saline intrusion |
| saline water |
| saturated soil |
| sea(\s\|-\|)level ris[a-z]* |
| severe climat[a-z]* |
| storm[a-z]* |
| tornado[a-z]* |
| tropical(\s\|-\|)storm[a-z]* |
| typhoon[a-z]* |
| uncertaint[a-z]* [[a-z]*\s{0,1}]{0,1}climat[a-z]* |
| uneven precipitation |
| water(\|-\|\s)logging |
| **Cultural Social Norms** |
| (?:^\|\W)cooperative [a-z]* |
| (?:^\|\W)cultur[a-z]*(?:$\|\W) |
| (?:^\|\W)practice(s\|)(?:$\|\W) |
| (?:^\|\W)tradition[a-z]* |
| colon[a-z]* identi[a-z]* |
| common[a-z]* unders[a-z]* |
| disconnect[a-z]* [[a-z]*\s{0,1}]{0,1} communit[a-z]* |
| indigeno(u\|)s practice(s\|) |
| interaction communit[a-z]* |
| practice [[a-z]*\s{0,1}]{0,1}province |
| rural life |
| soci[a-z]* standard(s\|) |
| social change |
| social expectation[a-z]* |
| social identi[a-z]* |
| social norm(s\|) |
| social[a-z]* [[a-z]*\s{0,1}]{0,1}influence[a-z]* |
| social[a-z]* [[a-z]*\s{0,1}]{0,1}norm[a-z]* |
| social[a-z]* [[a-z]*\s{0,1}]{0,1}value[a-z]* |
| socio(-\|\s\|)cultur[a-z]* |
| subjective norm(s\|) |
| traditional authorit[a-z]* |
| **Damage Experience** |
| asset[a-z]* damag[a-z]* |
| build[a-z]* damag[a-z]* |
| build[a-z]* vulnerab[a-z]* |
| collaps[a-z]* [[a-z]*\s{0,1}]{0,1}system(s\|) |
| crop failure(s\|) |
| damag[a-z]* [[a-z]*\s{0,1}]{0,1}propert[a-z]* |
| damag[a-z]* curv[a-z]* |
| damag[a-z]* experienc[a-z]* |
| de(\s\|-\|)forestation |
| food erosion |
| harvest loss |
| hous[a-z]* damag[a-z]* |
| infrast[a-z]* damag[a-z]* |
| intensification [[a-z]*\s{0,1}]{0,1}production |
| large damage |
| perceive(d\|) damage(s\|) |
| quality [[a-z]*\s{0,1}]{0,1}soil |
| soil [[a-z]*\s{0,1}]{0,1}erosion |
| soil infertil[a-z]* |
| soil quality |
| soil salin[a-z]* |
| vulnerab[a-z]* [[a-z]*\s{0,1}]{0,1}build[a-z]* |
| **Demographic Factors** |
| (?:^\|\W)black |
| (?:^\|\W)budd[a-z]* |
| (?:^\|\W)cast(?:$\|\W) |
| (?:^\|\W)child[a-z]* |
| (?:^\|\W)muslim[a-z]* |
| (?:^\|\W)race(?:$\|\W) |
| christian[a-z]* |
| coloni(s\|z)[a-z]* |
| colonialism |
| demograph[a-z] |
| ethnicit[a-z]* |
| family [[a-z]*\s{0,1}]{0,1}structure |
| hindu[a-z]* |
| household size |
| indigen[a-z]* group[a-z]* |
| indigen[a-z]* people |
| indigen[a-z]* person |
| latin[a-z]* |
| marital status |
| number [[a-z]*\s{0,1}]{0,1}family |
| people [[a-z]*\s{0,1}]{0,1}color |
| people [[a-z]*\s{0,1}]{0,1}race |
| population density |
| population grow[a-z]* |
| population pressure |
| population sensitive |
| reduc[a-z]* [[a-z]*\s{0,1}]{0,1}population |
| religion |
| religious |
| socio(-\|\s\|)[a-z]* |
| vulnerab[a-z]* [[a-z]*\s{0,1}]{0,1}population(s\|) |
| **Economic Factors** |
| (?:^\|\W)bankrupt[a-z]* |
| (?:^\|\W)inequalit[a-z]* |
| (?:^\|\W)market[a-z]* |
| (?:^\|\W)money |
| (?:^\|\W)povert[a-z]* |
| (?:^\|\W)production(s\|) |
| (?:^\|\W)productivit[a-z]* |
| (?:^\|\W)profit[a-z]* |
| access[a-z]* [[a-z]*\s{0,1}]{0,1}financ[a-z]* |
| access[a-z]* [[a-z]*\s{0,1}]{0,1}resource[a-z]* |
| access[a-z]* improv[a-z]* productivity |
| ad(a\|o)pt[a-z]* cost[a-z]* |
| agricult[a-z]* [[a-z]*\s{0,1}]{0,1}income[a-z]* |
| cost[a-z]* [[a-z]*\s{0,1}]{0,1}ad(a\|o)pt[a-z]* |
| cost[a-z]* [[a-z]*\s{0,1}]{0,1}crop[a-z]* |
| cost[a-z]* [[a-z]*\s{0,1}]{0,1}management[a-z]* |
| cost[a-z]* [[a-z]*\s{0,1}]{0,1}prod[a-z]* |
| crop[a-z]* [[a-z]*\s{0,1}]{0,1}production[a-z]* |
| crop[a-z]* [[a-z]*\s{0,1}]{0,1}productivit[a-z]* |
| distributi[a-z]* [[a-z]*\s{0,1}]{0,1}effect[a-z]* |
| econom[a-z]* [[a-z]*\s{0,1}]{0,1}dimension[a-z]* |
| econom[a-z]*(\|-)[a-z]* |
| economic challenge(s\|) |
| employment[a-z]* |
| expensive[a-z]* [[a-z]*\s{0,1}]{0,1}ad(a\|o)pt[a-z]* |
| food[a-z]* [[a-z]*\s{0,1}]{0,1}security |
| food[a-z]* [[a-z]*\s{0,1}]{0,1}storage[a-z]* |
| household[a-z]* [[a-z]*\s{0,1}]{0,1}capital[a-z]* |
| insufficient fund[a-z]* |
| lack [[a-z]*\s{0,1}]{0,1}capital |
| liquidity constrain[a-z]* |
| livelihood[a-z]* [[a-z]*\s{0,1}]{0,1}vulnerab[a-z]* |
| marginalized |
| market failure |
| market[a-z]* [[a-z]*\s{0,1}]{0,1}integration[a-z]* |
| market[a-z]* distance[a-z]* |
| mone[a-z]* resource(\|s) |
| performance [[a-z]*\s{0,1}]{0,1}expectanc[a-z]* |
| political [[a-z]*\s{0,1}]{0,1}trend |
| poor individual(s\|) |
| poor[a-z]* [[a-z]*\s{0,1}]{0,1}farm[a-z]* |
| poor[a-z]* [[a-z]*\s{0,1}]{0,1}household[a-z]* |
| production[a-z]* [[a-z]*\s{0,1}]{0,1}cost[a-z]* |
| social economic |
| subsistence [[a-z]*\s{0,1}]{0,1}agricultur[a-z]* |
| subsistence[a-z]* [[a-z]*\s{0,1}]{0,1}farm[a-z]* |
| unaffordable |
| vulnerab[a-z]* [[a-z]*\s{0,1}]{0,1}household[a-z]* |
| **Education** |
| (?:^\|\W)school(s\|) |
| ^literacy$ |
| educated |
| education |
| level [[a-z]*\s{0,1}]{0,1}education |
| level [[a-z]*\s{0,1}]{0,1}literacy |
| literacy level(s\|) |
| years [[a-z]*\s{0,1}]{0,1}school[a-z]* |
| **Farm Characteristics** |
| absence [[a-z]*\s{0,1}]{0,1}infrastructure(s\|) |
| household[a-z]* [[a-z]*\s{0,1}]{0,1}asset[a-z]* |
| labo(u\|)r(\|-\|\s)time |
| small(\s\|-\|)holder farm[a-z]* |
| small(\s\|-\|)product[a-z]* |
| small(\s\|-\|)scale farm[a-z]* |
| small[a-z]* [[a-z]*\s{0,1}]{0,1}farm[a-z]* |
| water [[a-z]*\s{0,1}]{0,1}consumption[a-z]* |
| water management[a-z]* |
| **Farm Size** |
| company size |
| farm size |
| farm[a-z]* land |
| farm[a-z]* plot |
| individual farmer |
| low [[a-z]*\s{0,1}]{0,1}farmer |
| **Farming Experience** |
| (?:^\|\W)abilit[a-z]* |
| (?:^\|\W)able(?:$\|\W) |
| (?:^\|\W)inability |
| (?:^\|\W)innovator(?:$\|\W) |
| (?:^\|\W)skill(s\|)(?:$\|\W) |
| ^experience(s\|)$ |
| capacity building |
| climat[a-z]* [[a-z]*\s{0,1}]{0,1}knowledge |
| develop[a-z]* [[a-z]*\s{0,1}]{0,1}knowledge |
| experimental knowledge |
| farm[a-z]* [[a-z]*\s{0,1}]{0,1}abilit[a-z]* |
| farm[a-z]* [[a-z]*\s{0,1}]{0,1}experience(s\|) |
| farm[a-z]* [[a-z]*\s{0,1}]{0,1}knowledge |
| farm[a-z]* [[a-z]*\s{0,1}]{0,1}skill[a-z]* |
| farm[a-z]* [[a-z]*\s{0,1}]{0,1}underst[a-z]* |
| farmer technical [a-z]* |
| human(\|-\|\s)capital |
| knowledge construction |
| knowledge develop[a-z]* |
| knowledge skill(s\|) |
| managerial capacit[a-z]* |
| present knowledge |
| self(\|-\|\s)sufficien[a-z]* |
| skill abilit[a-z]* |
| technical skill |
| years [[a-z]*\s{0,1}]{0,1}farming |
| **Gender** |
| (?:^\|\W)father(?:$\|\W) |
| (?:^\|\W)female(?:$\|\W) |
| (?:^\|\W)gender[a-z]*(?:$\|\W) |
| (?:^\|\W)girl[a-z]*(?:$\|\W) |
| (?:^\|\W)male(?:$\|\W) |
| (?:^\|\W)man(?:$\|\W) |
| (?:^\|\W)men(?:$\|\W) |
| (?:^\|\W)mother(?:$\|\W) |
| (?:^\|\W)woman(?:$\|\W) |
| (?:^\|\W)women(?:$\|\W) |
| gender[a-z]* differ[a-z]* |
| gender[a-z]* norm(\|s) |
| gender[a-z]* role(\|s) |
| **Government Support** |
| (?:^\|\W)law(s\|)(?:$\|\W) |
| (?:^\|\W)municipal[a-z]* |
| (?:^\|\W)program[a-z]* |
| (?:^\|\W)refund[a-z]* |
| ^government[a-z]*$ |
| ad(a\|o)pt[a-z]* polic[a-z]* |
| civil societ[a-z]* |
| coastal polic[a-z]* |
| compensation [[a-z]*\s{0,1}]{0,1}loss[a-z]* |
| design polic[a-z]* |
| emergency fund[a-z]* |
| environmental group(s\|) |
| establishment group(s\|) |
| food[a-z]* [[a-z]*\s{0,1}]{0,1}program[a-z]* |
| govern[a-z]* mechanism(s\|) |
| government[a-z]* |
| government[a-z]* [[a-z]*\s{0,1}]{0,1}aid[a-z]* |
| government[a-z]* [[a-z]*\s{0,1}]{0,1}bond |
| government[a-z]* [[a-z]*\s{0,1}]{0,1}loan(s\|) |
| government[a-z]* [[a-z]*\s{0,1}]{0,1}program(s\|) |
| government[a-z]* [[a-z]*\s{0,1}]{0,1}support(s\|) |
| high(-\|\s\|)level stakeholder(s\|) |
| institution[a-z]* [[a-z]*\s{0,1}]{0,1}factor |
| institution[a-z]* barrier(s\|) |
| institution[a-z]* constrain[a-z]* |
| institution[a-z]* intervent[a-z]* |
| institution[a-z]* norm(s\|) |
| institution[a-z]* role(s\|) |
| international [[a-z]*\s{0,1}]{0,1}organization(s\|) |
| international [[a-z]*\s{0,1}]{0,1}partner |
| international aid[a-z]* |
| lack [[a-z]*\s{0,1}]{0,1}polic[a-z]* |
| legislat[a-z]* |
| local government[a-z]* |
| mangrove plantation[a-z]* |
| national government[a-z]* |
| national polic[a-z]* |
| national strateg[a-z]* |
| ngo(s\|) |
| oppressive structure(s\|) |
| planned ad(a\|o)pt[a-z]* |
| polic[a-z]* will[a-z]* |
| policies |
| policies tailor |
| policy |
| policy actor(s\|) |
| policy design |
| policy respons[a-z]* |
| political [[a-z]*\s{0,1}]{0,1}organ[a-z]* |
| political [[a-z]*\s{0,1}]{0,1}stress |
| poor [[a-z]*\s{0,1}]{0,1}stakeholder(s\|) |
| provisioning service |
| public [[a-z]*\s{0,1}]{0,1}system[a-z]* |
| public ad(a\|o)pt[a-z]* |
| regional government[a-z]* |
| regulation[a-z]* |
| role [[a-z]*\s{0,1}]{0,1}institution[a-z]* |
| rural develop[a-z]* |
| stakeholder(s\|) engagement |
| stakeholder(s\|) participation |
| state govern[a-z]* |
| subsid[a-z]* |
| support[a-z]* |
| watershed [[a-z]*\s{0,1}]{0,1}program[a-z]* |
| worker institution |
| **Income** |
| (?:^\|\W)income |
| (?:^\|\W)wealth[a-z]* |
| [a-z]*(\s\|-\|)income |
| famil[a-z]* [[a-z]*\s{0,1}]{0,1}income[a-z]* |
| farm[a-z]* [[a-z]*\s{0,1}]{0,1}income[a-z]* |
| household[a-z]* [[a-z]*\s{0,1}]{0,1}income[a-z]* |
| income[a-z]* [[a-z]*\s{0,1}]{0,1}agricultur[a-z]* |
| income[a-z]* [[a-z]*\s{0,1}]{0,1}level(s\|) |
| income[a-z]* [[a-z]*\s{0,1}]{0,1}source[a-z]* |
| poor [[a-z]*\s{0,1}]{0,1}income |
| **Non-Climate Related Hazard** |
| (?:^\|\W)famine |
| death [[a-z]*\s{0,1}]{0,1}animal[a-z]* |
| death [[a-z]*\s{0,1}]{0,1}plant[a-z]* |
| disease(s\|) |
| fungi pest |
| non(-\|\s\|)climat[a-z]* factor(s\|) |
| shortage [[a-z]*\s{0,1}]{0,1}farm[a-z]* |
| sick[a-z]* animal[a-z]* |
| sick[a-z]* plant[a-z]* |
| **Owner** |
| (?:^\|\W)inherit[a-z]*(?:$\|\W) |
| (?:^\|\W)own[a-z]* |
| land(s\|-\|)owner(s\|) |
| **Psychological Barriers** |
| (?:^\|\W)anxiet[a-z]* |
| (?:^\|\W)denial(?:$\|\W) |
| climat[a-z]* [[a-z]*\s{0,1}]{0,1}concern[a-z]* |
| cognitive dissonance |
| concern[a-z]* [[a-z]*\s{0,1}]{0,1}climat[a-z]* |
| control[a-z]* [[a-z]*\s{0,1}]{0,1}behavi[a-z]* |
| depression |
| farm[a-z]* [[a-z]*\s{0,1}]{0,1}uncertain[a-z]* |
| feel[a-z]* [[a-z]*\s{0,1}]{0,1}guilt[a-z]* |
| lack[a-z]* [[a-z]*\s{0,1}]{0,1}option(s\|) |
| perceive(d\|) control |
| perceive(d\|) inabilit[a-z]* |
| psychological barrier(s\|) |
| psychological stress |
| scepticism |
| **Psychological Drivers** |
| (?:^\|\W)agency[a-z]*(?:$\|\W) |
| (?:^\|\W)autonom[a-z]* |
| (?:^\|\W)aware[a-z]* |
| (?:^\|\W)belief(s\|) |
| (?:^\|\W)confidence |
| (?:^\|\W)desire(?:$\|\W) |
| (?:^\|\W)fear[a-z]*(?:$\|\W) |
| (?:^\|\W)motivation(s\|) |
| (?:^\|\W)perception(s\|) |
| (?:^\|\W)risk(s\|) |
| (?:^\|\W)trust(?:$\|\W) |
| (?:^\|\W)worr[a-z]* |
| ^attitude(s\|)$ |
| concern[a-z]* [[a-z]*\s{0,1}]{0,1}risk |
| degree [[a-z]*\s{0,1}]{0,1}agency |
| farm[a-z]* [[a-z]*\s{0,1}]{0,1}trust |
| immense difficult[a-z]* |
| negative affect[a-z]* |
| outcome(\s\|-\|)efficacy |
| perceiv[a-z]* efficacy |
| perceive(d\|) probabilit[a-z]* |
| perceive(d\|) responsibilit[a-z]* |
| perceive(d\|) risk(s\|) |
| place attachment |
| positive emotion |
| psychological driver(s\|) |
| psychological factor |
| response(\s\|-\|)efficacy |
| risk averse |
| self(\s\|-\|)efficacy |
| situation assessment |
| social learning |
| **Social Capital** |
| (?:^\|\W)friend |
| (?:^\|\W)neighbo(u\|)r |
| (?:^\|\W)peer(?:$\|\W) |
| ^family$ |
| collective action(s\|) |
| community involvement |
| cousin(s\|) |
| household community |
| institution[a-z]* connection(s\|) |
| kinship |
| local area(s\|) |
| local farmer(s\|) |
| local initiative(s\|) |
| membership [[a-z]*\s{0,1}]{0,1}group |
| neighbo(u\|)r(s\|) |
| network relation[a-z]* |
| participation social |
| profession[a-z]* network(\|s) |
| relationship communit[a-z]* |
| rural communit[a-z]* |
| sibling(s\|) |
| social capital |
| social cohesion |
| social network(\|s) |
| social organization(s\|) |
| social participation |
| social re(-\|\s\|)organization |
| social relation[a-z]* |
| solidarity |
| viral adoption(s\|) |
| viral spread[a-z]* |
| viral uptake(s\|) |
| window [[a-z]*\s{0,1}]{0,1}opportunit[a-z]* |


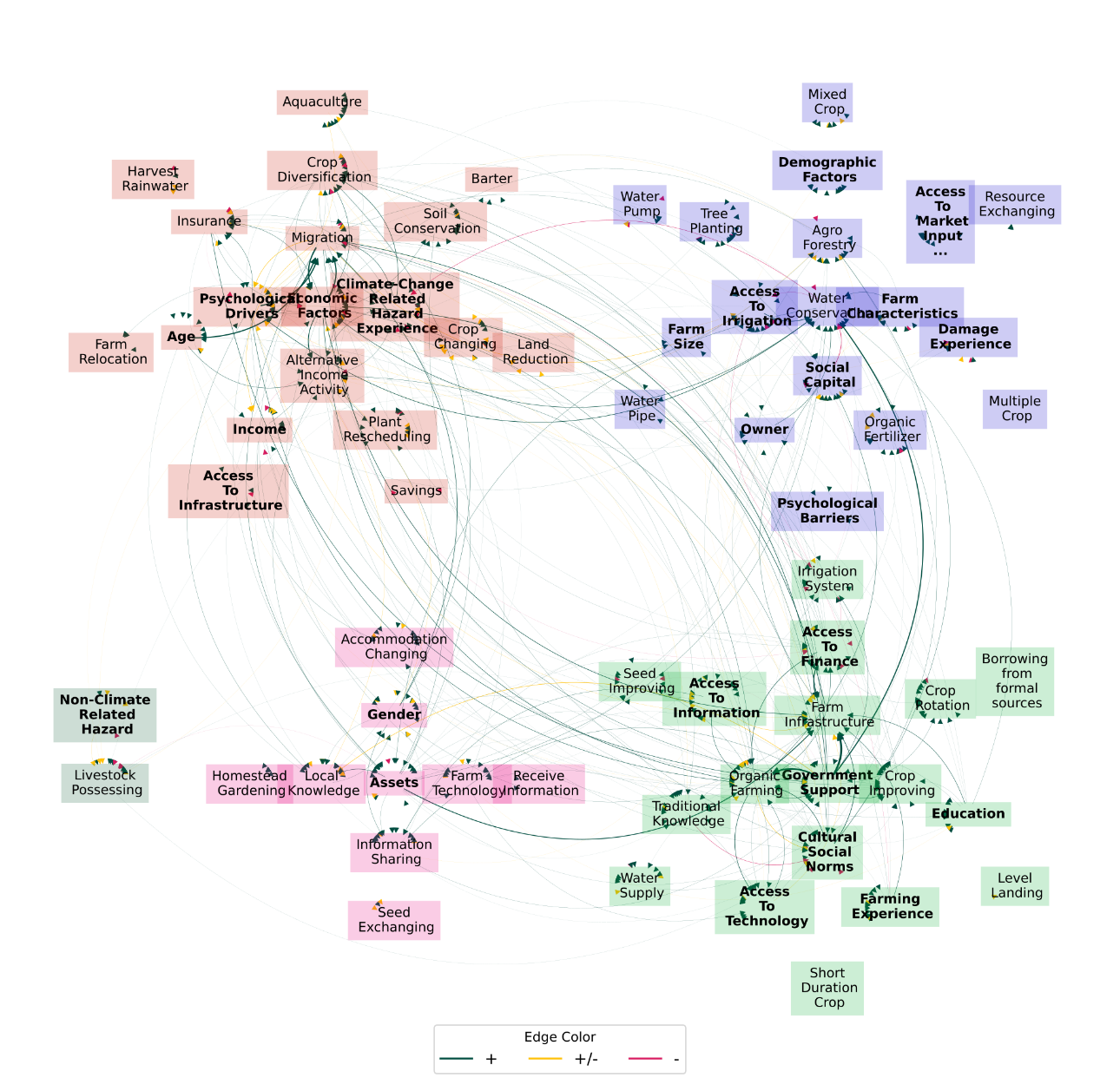


**Fig D1. Clustered network of farmers’ climate change adaptation measures and factors (in bold).**

**Table D2:** **Percentage of times the factors appear in the articles broken down by region.**

| Overall Group | Factors Categories | | Africa  N=100 | Asia  N=130 | Europe  N=30 | LAC  N=39 | Northern America  N=25 | Oceania  N=16 |
| --- | --- | --- | --- | --- | --- | --- | --- | --- |
| Adaptation Type | **Transformational** | | 38.1% | 37.9% | 36.6% | 36.2% | 30.3% | 33.3% |
| Hazard Experience | **Hazard Experience** | CC Related Hazard | 55.7% | 64.3% | 40.0% | 59.4% | 58.3% | 53.3% |
|  |  | Damage Experience | 19.3% | 13.4% | 6.7% | 12.5% | 4.2% | 6.7% |
|  |  | Non-CC Related Hazard | 17.0% | 14.3% | 13.3% | 18.8% | 8.3% | 6.7% |
| Individual AC | **Access to Infrastructure** | Access to Infrastructure | 9.1% | 6.3% | 3.3% | 3.1% | 0.0% | 0.0% |
|  |  | Access to Irrigation | 28.4% | 37.5% | 26.7% | 40.6% | 37.5% | 20.0% |
|  |  | Access to Market input output | 4.5% | 4.5% | 0.0% | 9.4% | 0.0% | 0.0% |
|  | **Age** | | 45.5% | 33.0% | 40.0% | 18.8% | 20.8% | 6.7% |
|  | **Assets** | | 69.3% | 52.7% | 63.3% | 50.0% | 50.0% | 53.3% |
|  | **Demographic Factors** | | 38.6% | 34.8% | 33.3% | 25.0% | 33.3% | 26.7% |
|  | **Economic Factors** | | 80.7% | 79.5% | 86.7% | 71.9% | 62.5% | 66.7% |
|  | **Education** | | 38.6% | 39.3% | 23.3% | 25.0% | 33.3% | 20.0% |
|  | **Farm Chars.** | Farm Characteristics | 27.30% | 28.60% | 20.00% | 21.90% | 8.30% | 6.70% |
|  |  | Farm Size | 17.00% | 14.30% | 10.00% | 3.10% | 12.50% | 6.70% |
|  | **Farming Experience** | | 46.6% | 37.5% | 43.3% | 37.5% | 37.5% | 53.3% |
|  | **Gender** | | 44.3% | 24.1% | 16.7% | 15.6% | 16.7% | 6.7% |
|  | **Income** | | 37.5% | 44.6% | 40.0% | 40.6% | 33.3% | 26.7% |
|  | **Owner** | | 25.0% | 18.8% | 20.0% | 18.8% | 25.0% | 20.0% |
| Institutional AC - Formal Institutions | **Access to Finance** | | 53.4% | 52.7% | 63.3% | 37.5% | 25.0% | 53.3% |
|  | **Government Support** | | 79.5% | 74.1% | 83.3% | 65.6% | 70.8% | 60.0% |
| Institutional AC - Informal Institutions | **Cultural Social Norms** | | 61.4% | 49.1% | 60.0% | 56.3% | 62.5% | 73.3% |
|  | **Social Capital** | | 43.2% | 35.7% | 43.3% | 28.1% | 20.8% | 33.3% |
| Knowledge and Information | **Access to Information** | | 53.4% | 41.1% | 36.7% | 34.4% | 41.7% | 40.0% |
| Psychological Factors | **Psychological Factors** | Psychological Barriers | 3.4% | 4.5% | 3.3% | 0.0% | 20.8% | 6.7% |
|  |  | Psychological Drivers | 70.5% | 67.9% | 73.3% | 53.1% | 75.0% | 73.3% |
| Technology | **Access to Technology** | | 31.8% | 39.3% | 43.3% | 43.8% | 29.2% | 33.3% |

**Note:** The total number of articles is above N=281 as some articles discussed countries from more than one region. AC Adaptive.
